# Effect of Sex on Intestinal Microbial Metabolites of Hainan Special Wild Boars

**DOI:** 10.3390/ani14152164

**Published:** 2024-07-25

**Authors:** Xiaozhe Wang, Qiong Wen, Hongfen Wu, Wenchuan Peng, Keqi Cai, Zhen Tan, Wei Na, Kebang Wu

**Affiliations:** 1 School of Tropical Agriculture and Forestry, Hainan University, Danzhou 571737, China; wxz202206@163.com (X.W.); 990815@hainanu.edu.cn (K.W.); 2 Sanya Institute, China Agricultural University, Sanya 572024, China; 3 State Key Laboratory of Animal Nutrition, Department of Companion Animal Science, China Agricultural University, Beijing 100193, China; 4 Wuhan Xiangda Feedstuff Co., Ltd., Wuhan 430045, China; 5 Key Laboratory of Animal Genetics, Breeding and Reproduction of Shaanxi Province, College of Animal Science and Technology, Northwest A&F University, Yangling 712100, China; 6 Shenzhen Branch, Guangdong Laboratory for Lingnan Modern Agriculture, Genome Analysis Laboratory of the Ministry of Agriculture and Rural Affairs, Agricultural Genomics Institute at Shenzhen, Chinese Academy of Agricultural Sciences, Shenzhen 518124, China

**Keywords:** Hainan special wild boar, metabolites, non-targeted metabolomics, gas chromatography, sex differences

## Abstract

**Simple Summary:**

Metabolites of intestinal microorganisms play an important role in the growth process of animals, and there are differences in the feeding behavior of animals of different sexes. The aim of this study was to reveal the sex differences in intestinal microbial metabolites of Hainan special wild boars. This study shows that the highest number of differential metabolites was found between entire males and females, the differential metabolites were enriched in more metabolic pathways, and castration reduced this difference. This study provides a certain metabolite database for future precision feeding of Hainan special wild boars and other pig breeds of different sexes.

**Abstract:**

The intestinal microbiota and its metabolites are essential for the health and growth development of animals. Current research indicates that sex has a certain impact on the structure and function of the intestinal microbiota, but there are few reports on sex differences in intestinal microbiota metabolites, including those of castrated male animals. This study aimed to explore the impact of sex on the intestinal microbial metabolites of Hainan special wild boars (10 entire male pigs, 10 female pigs, and 10 castrated male pigs, denoted EM, FE, and CM, respectively) by employing non-targeted metabolomics and gas chromatography. A total of 1086 metabolites were detected, with the greatest number of differential metabolites observed between EM and FE (54 differential metabolites, including 18 upregulated and 36 downregulated metabolites), the fewest between CM and FE (7 differential metabolites, including 1 upregulated and 6 downregulated metabolites), and an intermediate number between CM and EM (47 differential metabolites, including 35 upregulated and 12 downregulated metabolites). Differential metabolites were involved in more pathways between EM and FE and between CM and EM, including amino acid metabolism and digestive system pathways, whereas differential metabolites were involved in the fewest pathways between CM and FE. Correlation analysis showed *Ruminococcaceae UCG-009*, *uncultured_bacterium_o_SAR324_cladeMarine_group_B*, and *Candidatus Saccharimonas* contributed to the production of metabolites such as trehalose, docosatrienoic acid, D(−)-beta-hydroxy butyric acid, and acetyl-DL-leucine. The levels of acetic acid, propionic acid, butyric acid, isobutyric acid, valeric acid, and isovaleric acid were significantly higher in EM than in FE, with CM falling between the two. *Streptococcus*, *Lachnospiraceae_NK4A136_group* and *Rikenellaceae_RC9_gut_group* showed a significant positive correlation with the production of short-chain fatty acids (SCFAs), while *[Eubacterium]_coprostanoligenes_group*, *uncultured_bacterium_f_p–251–o5* and *Ruminococcaceae_UCG–005* showed a significant negative correlation with the generation of SCFAs. This study provides foundational data and significant insights into precision feeding strategies for Hainan special wild boars of different sexes, as well as the study of sex differences in intestinal microbial metabolites in animals.

## 1. Introduction

Intestinal microbiota is critical to host health, and the role of intestinal microbiota metabolites in maintaining and improving pig health should not be overlooked [1]. These metabolites may have positive or negative effects on animals under conditions of intestinal microbiota dysbiosis or stress, and the metabolites produced by intestinal microbiota can promote or inhibit the growth of certain microbes in the gastrointestinal tract [2]. Metabolic intermediates are involved in cellular functions and processes, such as energy metabolism, protein activity regulation, signal transduction, and defense mechanisms, which can be detected through metabolomic techniques [3]. Short-chain fatty acids (SCFAs) are the main products of intestinal microbiota fermentation of indigestible dietary fiber, including acetate, butyrate, propionate, and valerate [4,5], which represent the primary carbon flow from diet to host through microbes [6]. Short-chain fatty acids can significantly impact host health at the cellular, tissue, and organ levels by participating in intestinal barrier, glucose homeostasis, immune response, and obesity-related mechanisms [7]. In addition, lactic acid produced by lactic acid bacteria can be converted into SCFAs through a metabolite cross-feeding mechanism, and SCFAs can be transformed into branched-chain amino acids (BCAAs) by intestinal microbiota to produce energy [8]. Overall, the metabolites of intestinal microbiota play a key role in regulating intestinal health, immune function, metabolism, intestinal homeostasis, and cognitive function [1,2,3,4,5,6,7,8].

An increasing number of studies have shown that there is a profound interaction between the host’s sex and the intestinal microbiota, but the connection between metabolites and sex is also important [9,10]. Studies have shown that there are sex differences in the metabolites in serum and urine, with significant sex bias in the abundance of amino acids, lipids, and sugars in the serum [11]. In studies of mice and humans, sex differences have been repeatedly observed to affect the composition of metabolites in feces, serum, and bile [12,13,14,15,16]. For example, female mice exhibit higher serum concentrations of primary and secondary bile acids [12,13]. A study on human plasma metabolomics has shown that metabolic changes are related to sex differences in the aging process, with several metabolic pathways, including primary bile acid biosynthesis, lysine degradation, fatty acid biosynthesis, the pentose phosphate pathway, and linoleic acid metabolism, showing sex dimorphism [17]. About 33% of metabolites have significant differences between males and females, with increased levels of 5α-androstane-3β,17β-diol disulfate and BCAA isoleucine in males and increased creatine levels in females [18]. Clarifying sex-related biological differences is considered very valuable and will increasingly become a basic requirement for scientific research [3]. However, to this day, the relationship between sex-biased intestinal microbiota and metabolites has not been fully characterized.

Hainan special wild boar is a local hybrid breed in Hainan Province, China, that not only performs excellently in terms of edible value but also play a key role in the development of the local livestock industry and economic growth [19,20]. Feeding behavioral habits differ between sexes of pigs [21], but their metabolite basis is unclear. Based on the importance of metabolites of the intestinal microbiota for animal health, studying metabolite differences in Hainan special wild boars of different sexes may be an important strategy to improve livestock health and production performance. To our knowledge, few studies have compared the differences in metabolomics and SCFAs among entire males, females, and castrated males simultaneously.

One of our previous studies investigated the structure and function of intestinal microbiota in Hainan special wild boars of different sexes [19]. Metabolomics and gas chromatography were used to detect sex differences in metabolites produced by microorganisms of Hainan special wild boars in this study. This study will provide some basic data for the scientific feeding of Hainan special wild boars of different sexes, as well as some reference for the study of metabolite differences between animals of different sexes.

## 2. Materials and Methods

### 2.1. Animal Experiments

The Hainan special wild boars studied in this experiment were provided by the Yulvbao Wild Boar Breeding Center in Changjiang Li Autonomous County, Hainan Province, China. The study was conducted strictly in accordance with the Guidelines for Experimental Animals issued by the Ministry of Science and Technology (Beijing, China). The animal experiments in this study were approved by the Animal Welfare and Ethical Committee of Hainan University (permit HNUAUCC-2021-00003).

Prior to fattening, all pigs were fed the same diet under similar artificially controlled conditions. From fattening to sampling, all pigs were housed in the same fattening room and fed the same diet twice daily (Supplementary Table S1). They had free access to water, and no antibiotics were administered during the trial period. This study selected 30 healthy Hainan special wild boars, with the males being surgically castrated at 7 days of age. There were 10 EM, 10 FE, and 10 CM, designated as EM for entire (uncastrated) male pigs, FE for female pigs, and CM for castrated male pigs. All experimental pigs were sampled at the age of 8 months, with body weights ranging from approximately 45 to 55 kg.

### 2.2. Sample Collection

Sampling was conducted within two hours after the boars had finished their morning meal. Throughout the sampling process, masks and sterile gloves were worn at all times, and sterilized keys were used for sampling. Feces samples located at the center were collected immediately after the pigs defecated. After sampling, the feces samples were first collected in 2 mL centrifuge tubes, then immediately placed in liquid nitrogen for rapid freezing. Finally, the feces samples were transferred to a −80 °C refrigerator for freezing storage.

### 2.3. Sample Preparation and Metabolite Extraction

Metabolite extraction was referenced from a previous study and improved [22]. Briefly, all samples were thawed on ice, 500 μL of extract (mixture of methanol, acetonitrile, and ultrapure water in the ratio 2:2:1 (*V*/*V*)) was added to 25 mg of each sample, vortexed for 30 s, sonicated in an ice water bath for 5 min, incubated for 1 h at −40 °C, and centrifuged at 12,000 rpm for 15 min at 4 °C. The supernatant was pipetted into a clean centrifuge tube and subsequently dried under vacuum. The dried supernatant was dissolved in 400 μL of aqueous acetonitrile solution, vortexed for 30 s, sonicated for 10 min in an ice-water bath, and centrifuged at 13,000 rpm at 4 °C for 15 min. Then, 80 μL of supernatant was pipetted for liquid chromatography-mass spectrometry (LC-MS) analysis and another 10 μL of supernatant was taken from each test sample to prepare mixed quality control (QC) samples.

### 2.4. Metabolomic Analysis

The target compounds were chromatographed on a Waters Acquity UPLC BEH Amide (2.1 × 100 mm, 1.7 μm) liquid chromatography column using an Agilent 1290 (Santa Clara, CA, USA) ultrahigh-performance liquid chromatograph (UPLC). The liquid chromatographic phases A and B were aqueous and acetonitrile, respectively, and the aqueous phase contained 25 mmol/L ammonium acetate and 25 mmol/L ammonia. The gradient elution used was: 0–0.5 min, 95% B; 0.5–7 min, 95–65% B; 7–8 min, 65–40% B; 8–9 min, 40% B; 9–9.1 min, 40–95% B; 9.1–12 min, 95% B. The mobile phase flow rate was 0.5 mL/min, the column temperature was 25 °C, and the sample plate temperature was 4 °C. The injection volume was 4 μL for positive ions and 4 μL for negative ions.

A triple TOF 6600 high-resolution mass spectrometer was used for high-resolution mass spectrometry data acquisition in information-dependent acquisition (IDA) mode. In IDA mode, the data acquisition software (Analyst TF, version 1.7) automatically selects ions and acquires their secondary mass spectra based on the primary mass spectral data and predefined criteria. The 12 strongest ions with intensities greater than 100 were selected for secondary mass spectrometry in each cycle. The energy of collision-induced dissociation was 30 eV with a cycle time of 0.56 s. The parameters of the ion source were as follows: GS1: 60 psi, GS2: 60 psi, CUR: 35 psi, TEM: 600 °C, DP: 60 V, ISVF: 5000 V (Pos)/−4000 V(Neg).

### 2.5. Sample Preparation and SCFAs Detection

Short-chain fatty acids, including acetic, propionic, butyric, isobutyric, valeric, and isovaleric acids, were quantified using gas chromatography (GC) according to previous descriptions with some modifications [23]. Briefly, 300 μL of phosphoric acid solution and 1200 μL of ether (with 50 μg/mL 2-ethylbutyric acid as an internal standard) were added to 300 mg of sample, homogenized for 3 min, and centrifuged at 12,000 rpm at 4 °C for 10 min, then 800 μL of the supernatant was transferred to the injection vial for analysis using an HP-INNOWax column (30.0 m × 250 μm × 0.25 μm) and an Agilent 6890N (Santa Clara, CA, USA) gas chromatograph.

### 2.6. Data Processing and Statistical Analysis

The raw data were processed by Progenesis QI software (version 4.0), and identified based on the METLIN (Metabolite Link) database, KEGG (Kyoto Encyclopedia of Genes and Genomes) database and Biomark’s self-built library. The identified metabolites were synonymously compared in the KEGG database and annotated to the corresponding pathways [24]. Principal component analysis (PCA) was performed by the prcomp package. Metabolites with fold change greater than or equal to 2 and *p*-value less than 0.05 were selected as differential metabolites, Venn plots were constructed using a Venn software package (version 1.6), and metabolic pathway enrichment analysis was performed using the clusterProfiler software package (version 4.4.4) [25]. All statistical analyses for metabolomics were performed on the R platform (version 3.6.1). Differences between groups of SCFAs were analyzed by one-way ANOVA with Tukey’s multiple comparisons test and plotted using GraphPad Prism (version 9.3.0).

### 2.7. Correlation Analysis between Metabolomics, SCFAs, and Microorganisms

The V3 + V4 region of the 16S rRNA gene of the intestinal microorganisms of the boars was sequenced using the Illumina HiSeq 2500 platform as per one of our previous studies [19], and the correlation of metabolomics, SCFAs, and microorganisms in this study was analyzed using BMKCloud (www.biocloud.net, accessed on 22 June 2024). Data containing at least one correlation coefficient greater than 0.8 and correlation *p*-value less than 0.05 were retained for correlation analysis of metabolomics and microorganisms using the Hmisc package of the R platform (version 3.6.1). The correlation analysis between SCFAs and microorganisms (top ten genera in terms of abundance) was performed using Pearson’s correlation coefficient with a threshold of 0.1 and a significance *p*-value of 0.05.

## 3. Results

### 3.1. Metabolite Annotation and Principal Component Analysis

**Figure 1:**
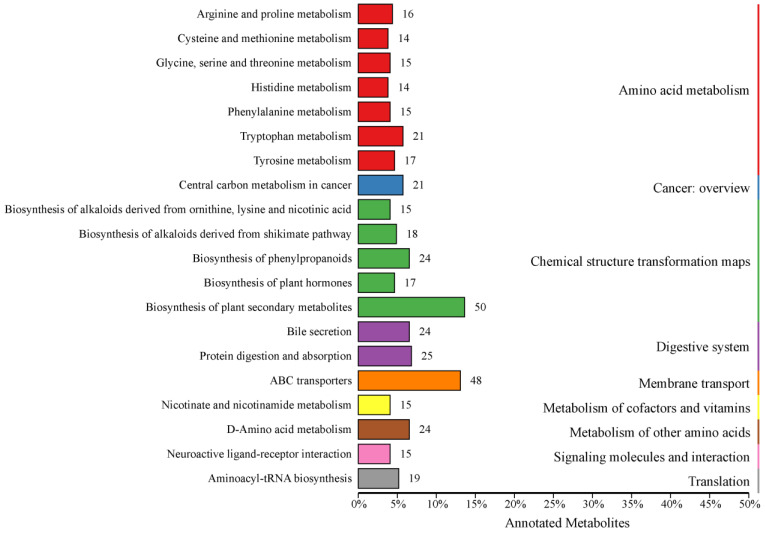
Kyoto Encyclopedia of Genes and Genomes database classification. Entries under the same box represent the hierarchical classification annotations of the KEGG pathways, corresponding to KO pathway level 1 and KO pathway level 2. The length of the bars indicates the number of metabolites annotated to each pathway.

The KEGG database facilitates researchers in studying genes, expression information and metabolite content as an integrated network [24], so all identified metabolites in this study were annotated using the KEGG database. A total of 1086 metabolites were identified, with 615 metabolites annotated in 242 categories (KO Pathway Level 2) in KEGG (Supplementary Table S2). Among the top 20 annotations with the most KO pathway level 2 entries, a total of 427 metabolites were annotated to 9 pathways (KO pathway level 1), which were amino acid metabolism, cancer—overview, chemical structure transformation maps, digestive system, membrane transport, metabolism of cofactors and vitamins, metabolism of other amino acids, signaling molecules and interaction, and translation (Figure 1).

At KO pathway level 1, amino acid metabolism, chemical structure transformation maps, and digestive system were found to be the pathways with the highest number of metabolites. The amino acid metabolism primarily consisted of seven secondary metabolic pathways, which were annotated with a total of 112 metabolites. The chemical structure transformation maps encompassed five secondary metabolic pathways, which were associated with 124 metabolites. The digestive system was characterized by two secondary metabolic pathways, which were linked to 49 metabolites.

At KO pathway level 2, the most abundant annotated metabolites were those involved in biosynthesis of plant secondary metabolites and ABC transporters, with 50 and 48, respectively. Furthermore, in excess of 20 metabolites had been assigned to metabolic pathways, including tryptophan metabolism, central carbon metabolism in cancer, biosynthesis of phenylpropanoids, bile secretion, protein digestion and absorption, and D-amino acid metabolism.

**Figure 2:**
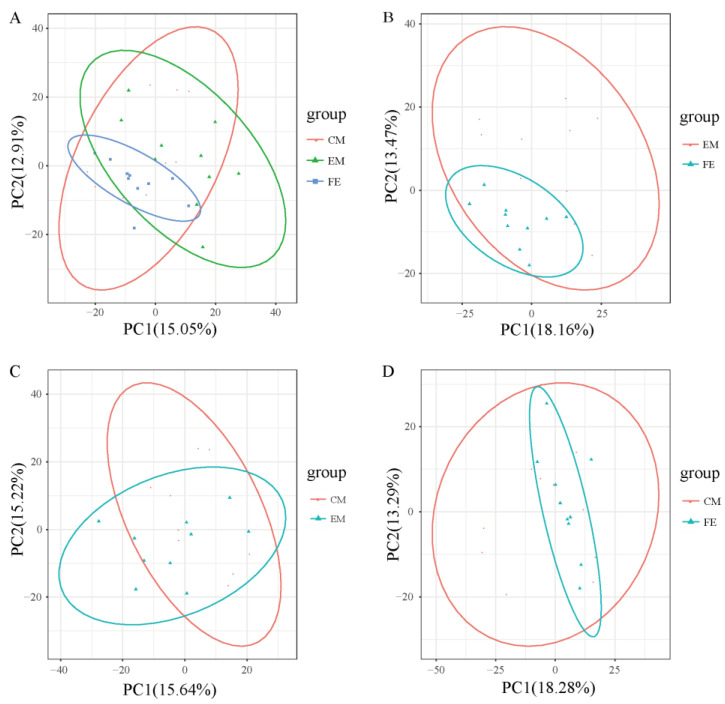
Principal component analysis. (**A**) Comparison of all groups. (**B**) Comparison between entire male pigs and female pigs. (**C**) Comparison between castrated male pigs and entire male pigs. (**D**) Comparison between castrated male pigs and female pigs. The *X*-axis represents the first principal component, and the *Y*-axis represents the second principal component, with the percentage on the axes indicating the contribution of each principal component to the variance among samples. Each point represents a sample, with samples from the same group depicted in the same color and samples from different groups distinguished by different colors.

Principal component analysis helps to preliminarily understand the overall metabolic differences between groups of samples and the degree of variation within the groups. It could be seen that the overall metabolic variation among FE samples was relatively low, while the overall metabolic variation among EM samples and CM samples was relatively higher (Figure 2). Moreover, the distribution range of metabolites in both EM samples and CM samples was broader than that in FE samples and the distribution range of metabolites in EM samples was closer to that in CM samples.

### 3.2. Analysis of Differential Metabolites and KEGG Functional Annotation

**Figure 3:**
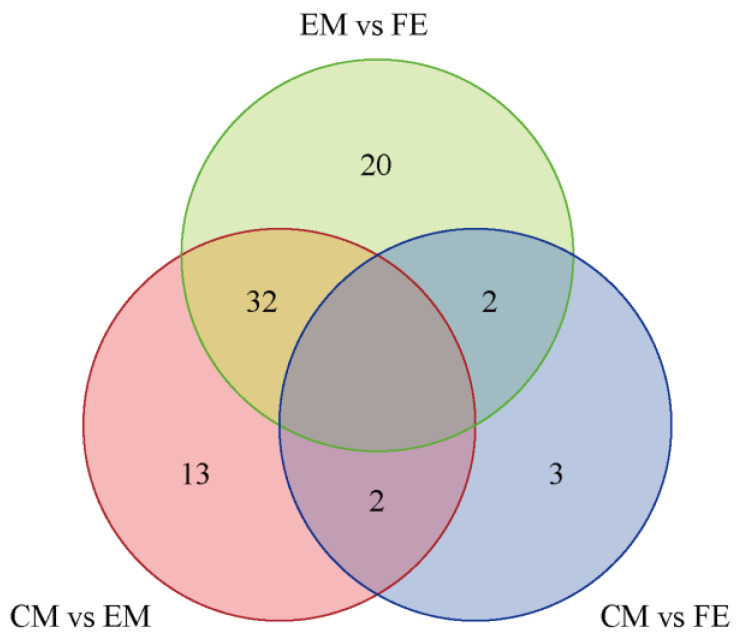
Differential metabolite Venn diagram. In the diagram, each circle represents a comparison group, with the numbers in the overlapping areas indicating the count of shared differential metabolites between the groups and the numbers in the non-overlapping areas indicating the count of unique differential metabolites for each group.

Based on the Venn diagram, the intersection and union relationships of differential metabolites between groups can be compared and analyzed. The greatest number of differential metabolites were identified between EM and CM (54 metabolites), the fewest differential metabolites were identified between CM and FE (7 metabolites), and there were 47 differential metabolites between CM and EM (Figure 3). A total of 32 differential metabolites exhibited identical values between EM vs. FE and CM vs. EM. Two differential metabolites exhibited identical values between EM vs. FE and CM vs. FE, as well as between CM vs. EM and CM vs. FE. Furthermore, 20 distinct differential metabolites were observed between EM and FE, 13 between CM and EM, and only 3 between CM and FE. All metabolites that differed between groups are included in Table 1.

A total of 18 differential metabolites were upregulated and 36 downregulated between EM and FE (Table 2). In the comparison between CM and EM, 35 differential metabolites were observed to be upregulated, while 12 were observed to be downregulated. Between CM and FE, there was one upregulated differential metabolite and six downregulated differential metabolites. The differential metabolites between EM and FE were primarily composed of organic acids, fatty acids and derivatives, amino acid derivatives, and cholesterol and steroids. In contrast, the differential metabolites observed between CM and FE were predominantly concentrated around amino acids and derivatives, organic acids, fatty acids, nucleosides, and nucleotide analogues. In addition, organic acids and amino acid derivatives were mainly included between CM and FE.

**Figure 4:**
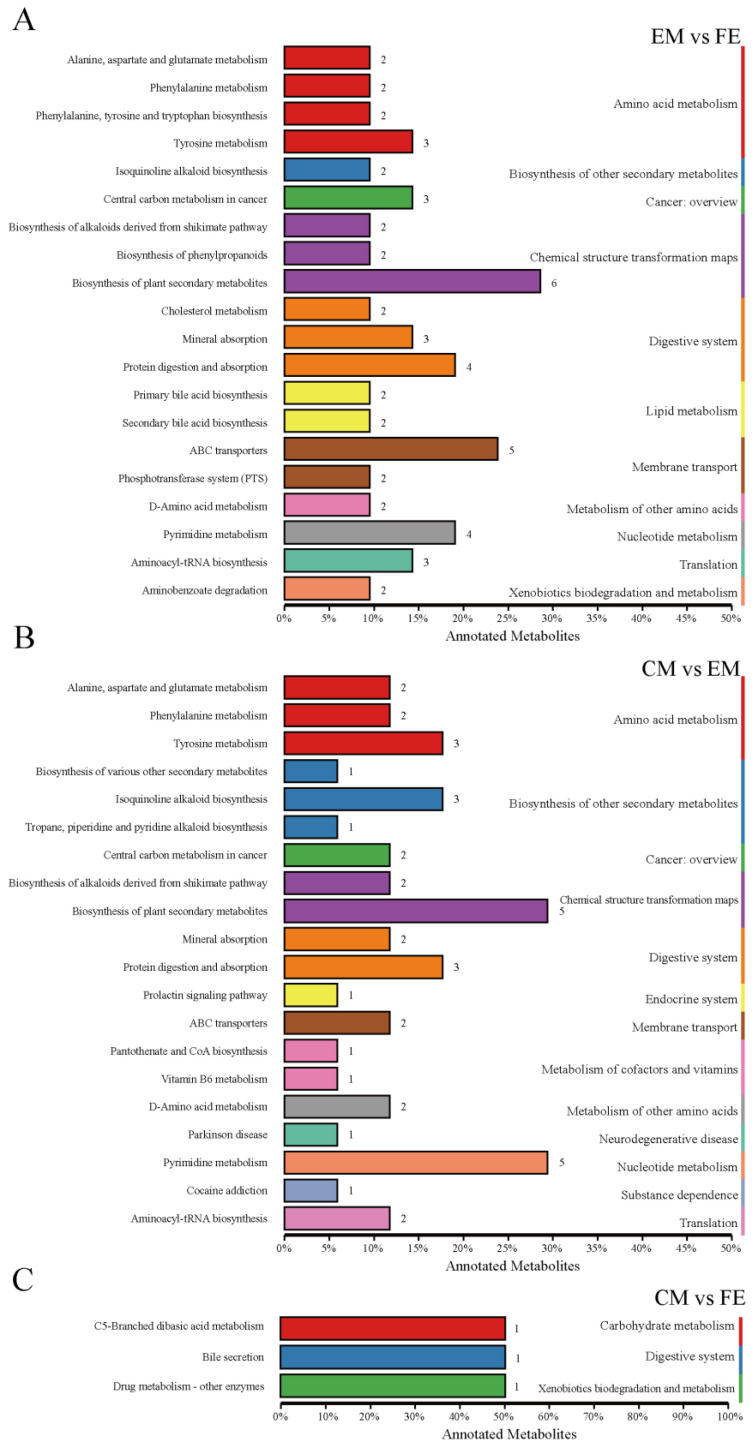
Classification of differential metabolite pathways. (**A**) Comparison between entire male pigs and female pigs. (**B**) Comparison between castrated male pigs and entire male pigs. (**C**) Comparison between castrated male pigs and female pigs. The top 20 metabolic pathways in relation to differential metabolites are illustrated in this graph. Different-colored bars represent the hierarchical classification annotations of the KEGG pathways, corresponding to the KO pathway level and the KEGG pathway name. The length of the bars indicates the number of differential metabolites annotated to each pathway.

Differential metabolites interact with each other in organisms to form different pathways, and analyses of metabolic pathways annotated as differential metabolites can lead to a more comprehensive and systematic understanding of the mechanisms of altered biological processes and trait development [25]. At KO pathway level 1, the EM vs. FE and CM vs. EM groups exhibited the greatest degree of metabolic pathway annotation, with 55 metabolites annotated to 11 pathways and 42 metabolites annotated to 13 pathways, respectively (Figure 4A,B). Of these, nine pathways were identical. The metabolic pathways of amino acid metabolism, chemical structure transformation maps, and digestive system exhibited the greatest number of annotated metabolites. Conversely, the CM vs. FE group had the fewest metabolic pathways annotated, including only three pathways annotated by three metabolites (Figure 4C). The CM vs. EM group exhibited four distinct metabolic pathways, namely, the endocrine system, metabolism of cofactors and vitamins, neurodegenerative disease, and substance dependence. In contrast, the EM vs. FE and CM vs. FE groups demonstrated a single metabolic pathway each, namely, lipid metabolism and carbohydrate metabolism, respectively.

At KO pathway level 2 of the comparison between EM and FE, the highest number of differential metabolites were annotated to the following categories: biosynthesis of plant secondary metabolites (six metabolites), ABC transporters (five metabolites) and protein digestion and absorption (four metabolites). Furthermore, two metabolites were identified in both the primary and secondary bile acid biosynthesis pathways. Five differential metabolites were concentrated in the pathways of biosynthesis of plant secondary metabolites and pyrimidine metabolism between CM and EM. Additionally, three further differential metabolites were concentrated in tyrosine metabolism, isoquinoline alkaloid biosynthesis, and protein digestion and absorption. Supplementary Table S3 shows the pathways with differential metabolite enrichment between subgroups based on the KEGG database.

### 3.3. Metabolomic and Microbiome Correlation Analysis

**Figure 5:**
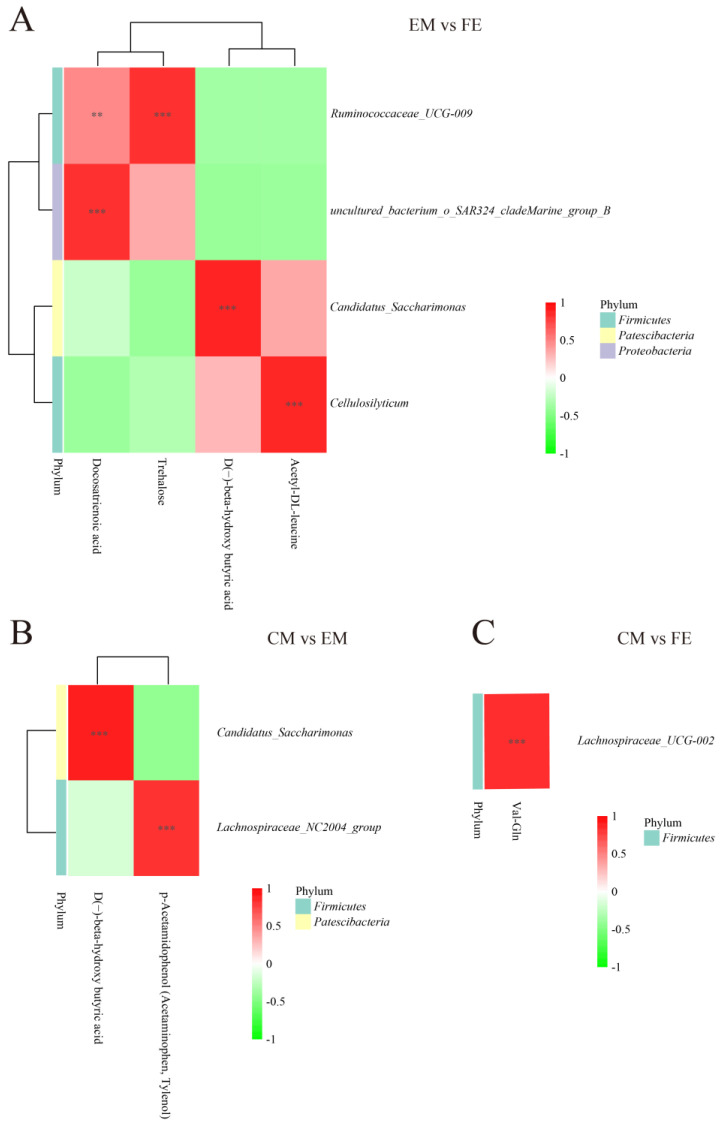
Correlation analysis of differential metabolites and differential microbiota. (**A**) Comparison between entire male pigs and female pigs. (**B**) Comparison between castrated male pigs and entire male pigs. (**C**) Comparison between castrated male pigs and female pigs. On the right are the differential microbes at the genus level, and at the bottom are the differential metabolites, with the dendrograms for the differential microbiota and metabolites on the left and top, respectively. Different colors represent the magnitude of the Pearson correlation coefficient, with an absolute value closer to 1 indicating a higher degree of correlation. Red indicates positive correlation, while green indicates negative correlation, and the darker the color, the stronger the correlation. An asterisk indicates a significant correlation between differential metabolites and differential microbiota, with ** and *** indicating increasing levels of significance, representing *p* < 0.01 and *p* < 0.001, respectively.

To understand the connection between differential metabolites and microorganisms, a correlation analysis was conducted. Correlations between metabolites and microorganisms were observed to be more extensive between EM and FE, with a lesser extent observed between CM and EM. Between EM and FE, *Ruminococcaceae UCG-009* showed a significant positive correlation with docosatrienoic acid and trehalose. The *Uncultured_bacterium_o_SAR324_cladeMarine_group_B*, *Candidatus_Saccharimonas* and *Cellulosilyticum* were found to be positively correlated with docosatrienoic acid, D(−)-beta-hydroxy butyric acid, and acetyl-DL-leucine, respectively (Figure 5A). In the CM vs. EM group, *Candidatus_Saccharimonas* exhibited a positive correlation with D(−)-beta-hydroxy butyric acid, while *Lachnospiraceae_NC2004_group* demonstrated a positive correlation with p-Acetamidophenol (Acetaminophen, Tylenol) (Figure 5B). There were fewer correlations between metabolites and microbial presence between CM and FE, and only *Lachnospiraceae_UCG-002* was positively correlated with Val-Gln (Figure 5C).

### 3.4. Short-Chain Fatty Acid Analysis

**Figure 6:**
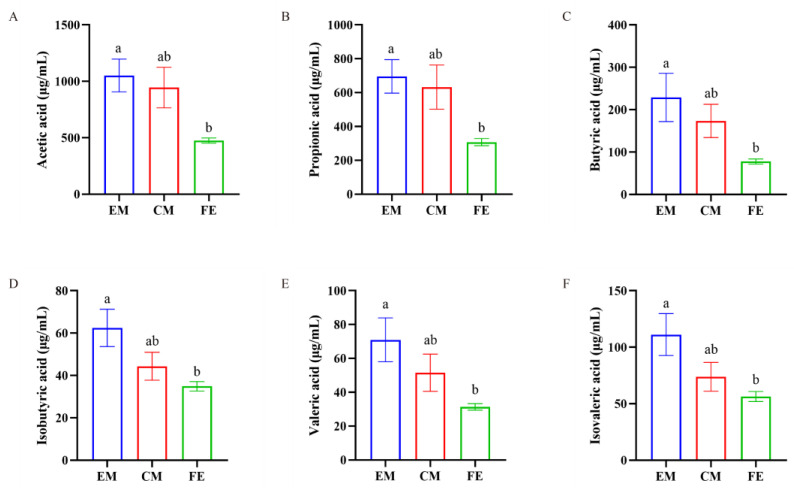
Comparison of SCFAs. (**A**) Acetic acid. (**B**) Propionic acid. (**C**) Butyric acid. (**D**) Isobutyric acid. (**E**) Valeric acid. (**F**) Isovaleric acid. Significant differences are denoted by different lowercase letters (*p* < 0.05), all data are expressed as means ± SEM (*n* = 8–10, individual extreme data points were eliminated).

Upon detection of SCFAs in the three groups of pigs, we found that the SCFAs in EM, FE, and CM were predominantly acetic acid, propionic acid, and butyric acid, with relatively lower levels of isobutyric acid, valeric acid, and isovaleric acid (Figure 6). Additionally, the levels of acetic acid, propionic acid, butyric acid, isobutyric acid, valeric acid, and isovaleric acid in EM were significantly higher than those in FE (*p* < 0.05), while the levels in CM were between those of EM and FE.

### 3.5. Short-Chain Fatty Acid and Microbiome Correlation Analysis

**Figure 7:**
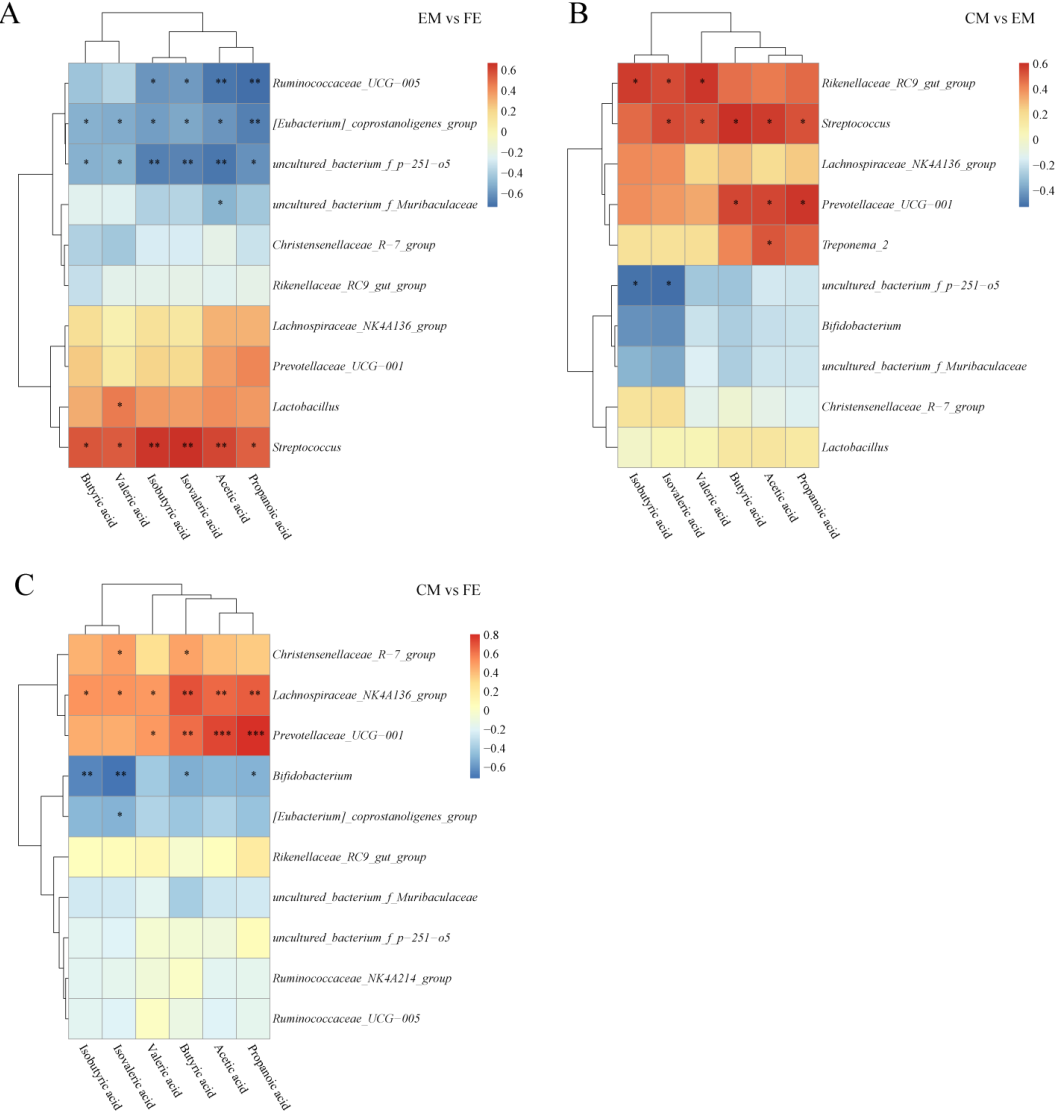
Correlation analysis of SCFAs and microbiota. (**A**) Comparison between entire male pigs and female pigs. (**B**) Comparison between castrated male pigs and entire male pigs. (**C**) Comparison between castrated male pigs and female pigs. On the right are the differential microbes at the genus level and at the bottom are the differential SCFAs, with the dendrograms for the differential microbiota and SCFAs at the left and top, respectively. Different colors represent the magnitude of the Pearson correlation coefficient, with an absolute value closer to 1 indicating a higher degree of correlation. Red indicates positive correlation, blue indicates negative correlation, and the darker the color, the stronger the correlation. An asterisk indicates a significant correlation between differential SCFAs and differential microbes, with *, **, and *** indicating increasing levels of significance, representing *p* < 0.05, *p* < 0.01, and *p* < 0.001, respectively.

To explore the relationship between SCFAs and microorganisms, a correlation analysis was conducted. A positive relationship was observed between SCFAs and *Streptococcus* and a negative relationship with *uncultured_bacterium_f_p–251–o5* and *[Eubacterium]_coprostanoligenes_group* when comparing EM and FE (Figure 7A). Furthermore, a positive correlation was observed between *Lactobacillus* and valeric acid, while a negative correlation was found between *Ruminococcaceae_UCG–005* and isobutyric acid, isovaleric acid, acetic acid, and propanoic acid. Between CM and EM, SCFAs other than isobutyrate showed a positive correlation with *Streptococcus*, while *uncultured_bacterium_f_p–251–o5* correlated with isobutyrate and isovalerate inversely (Figure 7B). In addition, *Rikenellaceae_RC9_gut_group* was proportional to isobutyric acid, isovaleric acid, and valeric acid, and *Prevotellaceae_UCG–001* was proportional to butyric acid, acetic acid, propanoic acid. Unlike the EM vs. FE and CM vs. EM groups, *Lachnospiraceae_NK4A136_group*, *Prevotellaceae_UCG–001*, and *Bifidobacterium* were the main genera correlated with SCFAs in the CM vs. FE group (Figure 7C). *Lachnospiraceae_NK4A136_group* and *Prevotellaceae_UCG–001* were positively correlated with SCFAs, while *Bifidobacterium* was negatively correlated with SCFAs.

## 4. Discussion

The impact of sex on metabolic differences in organisms is receiving widespread attention from scientists [26,27]. To our knowledge, this is the first study to compare the metabolomics and SCFAs of entire male pigs, female pigs, and castrated male pigs simultaneously. Intestinal microbiota and their metabolites have a profound impact on the growth performance, feed conversion efficiency, and health status of pigs [1]. Metabolites are the basis of an organism’s phenotype and can help us to understand biological processes and their mechanisms more intuitively and effectively. Hormonal patterns in pigs play an important role in metabolic profiles and can influence physiological changes in production performance and carcass traits [28]. It is common in animal husbandry to castrate male animals to reduce odors in pork, and castration leads to lower levels of sex hormone production in males and therefore reduces the metabolic differences between males and females [29,30]. As in a previous study [31], this study also showed that castrated male pigs are closer to female pigs in terms of metabolomics.

Many metabolite-based feed additives can be used to improve pig performance and health, potentially leading to higher economic returns [32]. Differential metabolites for Hainan special wild boars of different sexes mainly include amino acids and derivatives, nucleosides and nucleotides, organic acids, and fatty acids. Amino acid supplementation in the diet has been shown to regulate gene expression, reduce excess body fat, increase gut and skeletal muscle growth, and improve immunity and gut health [33,34]. Dietary nucleotides help to improve intestinal iron absorption, influence the metabolism of lipoproteins and long-chain polyunsaturated fatty acids, have a nutritive effect on the intestinal mucosa and liver, reduce the incidence of diarrhea, and promote the growth and maturation of intestinal epithelial cells [35]. Organic acids have a beneficial effect in lowering gastric pH, inhibiting the growth of pathogens, acting as a source of energy during intermediate metabolism in the gastrointestinal tract, increasing apparent digestibility throughout the tract, and improving growth performance [36]. Fatty acids are important components in the formation of lipids and contribute to energy metabolism, cell membrane stability, and the regulation of cellular processes [37]. Thus, differences in metabolites may be a partial explanation for differences in growth performance between Hainan special wild boars of different sexes [30]. In addition, our previous study on the α-diversity of the intestinal microbiota of Hainan special wild boars showed that female pigs have the highest intestinal microbial diversity, while entire male pigs have the lowest [19]. The large difference in intestinal microbial α-diversity between entire male pigs and female pigs is highly consistent with the largest number of differential metabolites between entire male pigs and female pigs in this study.

KEGG functional annotation helps to understand in which pathways the different metabolites are mainly distributed [25]. The differential metabolites between entire male pigs and female pigs were mainly involved in protein digestion and absorption and cholesterol metabolism in the digestive system. The absorption and digestion of protein are crucial for the growth and development of pigs. At the same developmental stage, the growth rate and body weight of entire male pigs are generally higher than those of female pigs. The differential metabolites involved in protein absorption and digestion between entire male pigs and female pigs may result in a higher growth rate and body weight of entire male pigs compared to female pigs. In the reproductive physiology of pigs, cholesterol is particularly crucial for the synthesis of sex hormones, affecting reproductive health and breeding performance [38], so the difference in cholesterol metabolism may be an important factor in sex differences between entire male pigs and female pigs. Meanwhile, cholesterol is a precursor for the biosynthesis of bile acids in living organisms [39], and bile acid content and metabolism differ between males and females [40,41]. Therefore, differences in primary and secondary bile acid biosynthesis in lipid metabolism pathways between entire male pigs and female pigs may correlate with differences in cholesterol metabolism [39]. The endocrine system pathway was a unique pathway annotated with differential metabolites between castrated male pigs and entire male pigs. Entire male pigs have an intact reproductive system and are capable of producing androgens, and altered levels of sex hormone production after castration may be an important reason for the significant differences in endocrine function between castrated male pigs and entire male pigs [42]. The difference in the digestive system metabolic pathway between castrated male pigs and female pigs includes bile secretion. Bile acids are a key component of bile, produced by the metabolism of cholesterol in the liver. Farnesoid X receptor (FXR) and Takeda G protein-coupled receptor 5 (TGR5) are important receptors in bile acid metabolism. Some studies have shown that intestinal microbiota may directly affect fertility and reproductive ability through the FXR and TGR5 receptors, possibly through the interaction between bile acids and sex hormones [43,44]. Bile acids can also affect the levels of testosterone in the plasma, which may in turn affect the host’s fertility [45,46]. However, it is currently unclear whether bile acids have an impact on female reproductive hormone levels and fertility.

In recent years, there has been an increase in research into animal metabolomics and microbiomics, and metabolite–microbe correlation analyses can help to further explore and investigate microbial drivers of metabolic change [47]. The results of this study demonstrated that docosatrienoic acid, D(−)-beta-hydroxybutyric acid, acetyl-DL-leucine, Val-Gln and *Ruminococcaceae_UCG-009*, *Candidatus_Saccharimonas*, *Cellulosilyticum*, and *Lachnospiraceae_UCG-002* were highly correlated. In addition, a previous study has shown that exogenous addition of organic acids to feed improves the growth performance of fattening pigs and has a significant effect on pig growth and development [48]. The present study suggests that regulating animal growth by adding specific metabolites such as fatty acids or amino acids to the diet may also be an important feeding strategy.

Undigested fats and carbohydrates in the gut, after microbial action, can produce a series of SCFAs, which in turn affect the growth and development of the body through various pathways [49]. For example, the acidic environment created by SCFAs in the gut can affect the production of pathogens and thus promote intestinal development [8], and the proliferation and differentiation of epithelial cells in the gut are also affected by SCFAs [1]. In recent years, many studies have confirmed the role of SCFAs in maintaining the intestinal health of pigs, especially butyrate, propionate, and acetate [50]. Butyrate and propionate, histone deacetylase inhibitors, can affect the host’s immune response and have anti-inflammatory and immunosuppressive effects, so they are considered to improve intestinal health [51]. In addition, butyrate, the main energy source for colon cells, has anti-inflammatory effects and maintains energy metabolism and homeostasis [52,53]. Butyrate can also restore beneficial microbes and reduce the metabolites of toxic microbes [54]. Acetate, which exists at high concentrations in peripheral circulation, usually has beneficial metabolic effects on white adipose tissue (WAT), the brain, and the liver. In WAT, increased acetate levels are associated with reduced lipolysis and reduced fat accumulation mediated by insulin. As a key receptor for acetate, G-protein-coupled receptor 43 (GPR43) has been shown to be related to leptin secretion, adipogenesis, and anti-lipolytic activity in WAT [55,56,57]. The highest level of acetate in this study was observed in samples from entire males and may be a key reason for their faster weight gain. The addition of SCFAs to feed regulates the gastrointestinal health, host immune function, and general welfare of animals, and may also serve as an energy source for bacteria [48].

Various intestinal microbes have been reported to influence the production of SCFAs and also to modulate the production of vitamins in the body, such as *Lactobacillus* and *Prevotella* [58,59]. The content of all SCFAs is highest in entire male pigs and lowest in female pigs, which is exactly the opposite of the richness and diversity in the α-diversity analysis of intestinal microbiota [19], possibly because SCFAs are produced by certain or a few types of microbes. Previous studies have shown that *Lachnospiraceae* and *Ruminococcaceae* can increase the concentration of butyrate and regulate intestinal homeostasis and inhibit pro-inflammatory cytokines by stimulating cell proliferation [60], while *Clostridium*, *Propionibacterium*, *Streptococcus*, and *Bacteroides* are involved in the production of branched-chain fatty acids (BCFAs) [61]. In this study, the production of SCFAs was significantly positively correlated with *Streptococcus*, *Lachnospiraceae_NK4A136_group*, *Rikenellaceae_RC9_gut_group*, *Prevotellaceae_UCG–001*, *Christensenellaceae_R–7_group*, and *Lactobacillus* and significantly negatively correlated with *[Eubacterium]_coprostanoligenes_group*, *uncultured_bacterium_f_p–251–o5*, *Ruminococcaceae_UCG–005*, and *Bifidobacterium*, and further studies are needed to confirm this.

Due to differences in feeding behavior between the sexes, specific feeding techniques may be required [21,62]. Metabolic processes, health, and productivity can be specifically improved by adding multiple metabolites or SCFAs to pig feed [32,48]. However, relatively few studies have been conducted on metabolites and SCFAs in pigs of different sexes. Future studies should focus more on the effects of supplementation of SCFAs or other metabolites on pigs of different sexes, which will help us to better understand the application of precision feeding in animal husbandry [63].

## 5. Conclusions

In short, our study revealed the existence of sex-based differences in the metabolites of intestinal microorganisms, both in terms of species and content, in Hainan special wild boars. The most notable discrepancies were observed between entire male pigs and female pigs, with male castration serving to narrow these differences. The metabolic pathways involved in differential metabolites in the intestinal microbiota of Hainan special wild boars differed between sexes, with the fewest differential metabolites involved in pathways between castrated male pigs and female pigs. In conclusion, this study provides an important reference for the precision feeding of Hainan special wild boars and other pig breeds of different sexes and for the study of differences in intestinal microbial metabolites in animals of different sexes.

## Figures and Tables

**Table 1:** Differential metabolites in different subgroups. “1” means it is a differential metabolite for the group and “0” means it is not a differential metabolite for the group.

Metabolite Name	EM vs. FE	CM vs. EM	CM vs. FE
Butabarbital	1	0	1
4,6-Diamino-5-formamidopyrimidine	0	1	0
Glycochenodeoxycholate	1	0	0
N-.alpha.-Acetyl-L-arginine	1	0	0
Actinonin	1	0	1
5-Hydroxyhexanoic acid	1	0	0
Methacholine	1	0	0
Cortisol 21-acetate	1	1	0
Inosine	1	0	0
L-Alanine	1	1	0
3,3′,4,5′-Tetrahydroxy-trans-stilbene	1	0	0
Docosatrienoic Acid	1	1	0
1-O-Octadecyl-sn-glyceryl-3-phosphorylcholine	1	0	0
p-Acetamidophenol (Acetaminophen, Tylenol)	1	1	0
Ser-Leu	1	0	0
N2-Acetyl-L-ornithine	0	1	0
3-Hydroxyanthranilic acid	1	0	0
Stearoylcarnitine	0	1	0
Cytidine	1	1	0
DL-lactate	1	1	0
Uracil	1	1	0
5-Methylcytosine	0	1	0
3-Methylglutaric acid	1	0	0
L-Leucine	1	0	0
L-Asparagine	1	1	0
1-Palmitoyl-sn-glycero-3-phosphocholine	1	1	0
Procaine	0	1	0
sn-Glycerol 1-phosphate	0	0	1
L-Dopa	1	1	0
Tyramine	0	1	0
N6-Methyladenine	1	1	0
1-Stearoyl-sn-glycerol 3-phosphocholine	1	1	0
2-Methyl-3-hydroxybutyric acid	1	1	0
trans-3-Coumaric acid	0	1	0
Thymine	1	1	0
6-Mercaptopurine	0	0	1
2-Hydroxyadenine	0	1	0
Hydroxyisocaproic acid	1	1	0
Phenylpyruvate	1	1	0
Glycolithocholic acid	1	0	0
Trehalose	1	0	0
Acetohydroxamic acid	0	1	0
5-Amino-4-carbamoylimidazole (AICA)	0	1	0
Oxyquinoline	1	1	0
D(−)-beta-hydroxy butyric acid	1	1	0
N-Tigloylglycine	0	0	1
Acetylglycine	1	1	0
Itaconic acid	0	1	1
L-Ascorbic acid	1	1	0
7-Oxocholesterol	1	1	0
Acetyl-DL-Leucine	1	1	0
3-Dehydroshikimic acid	1	0	0
Pyridoxine	0	1	0
PS(16:0/16:0)	1	1	0
Deoxygalactonojirimycin	0	1	0
3,3-Dimethylglutaric acid	1	0	0
Gaboxadol	0	1	0
D-Fructose	1	1	0
Val-Gln	0	1	1
Glycodeoxycholic acid	1	0	0
Phenol	1	0	0
N-Acetyl-L-phenylalanine	1	1	0
4-Hydroxycinnamic acid	1	1	0
Hydroxyphenyllactic acid	1	1	0
Scytalone	1	1	0
Glycocholic Acid	1	0	0
Pseudouridine	1	1	0
2-Dehydro-3-deoxy-D-gluconate	1	1	0
1-Methylxanthine	1	0	0
Androsterone sulfate	1	1	0
1-Stearoyl-2-hydroxy-sn-glycero-3-phosphocholine	1	1	0
Dihydroxyfumarate	1	0	0

**Table 2:** Up- and downregulated differential metabolites between different groups.

Metabolite Name	log_2_(Fold Change)	*p*-Value	Regulated
**EM vs. FE**			
1-Palmitoyl-sn-glycero-3-phosphocholine	1.352	<0.001	up
p-Acetamidophenol (Acetaminophen, Tylenol)	1.251	<0.001	up
Trehalose	1.252	<0.001	up
Glycochenodeoxycholate	1.973	0.001	up
1-Stearoyl-2-hydroxy-sn-glycero-3-phosphocholine	1.366	0.001	up
Cytidine	1.193	0.001	up
Docosatrienoic Acid	1.651	0.002	up
Glycocholic Acid	1.213	0.002	up
3-Hydroxyanthranilic acid	1.013	0.002	up
1-Stearoyl-sn-glycerol 3-phosphocholine	1.149	0.004	up
N-.alpha.-Acetyl-L-arginine	1.258	0.005	up
1-O-Octadecyl-sn-glyceryl-3-phosphorylcholine	1.120	0.005	up
7-Oxocholesterol	1.194	0.005	up
D-Fructose	1.057	0.007	up
Glycodeoxycholic acid	2.216	0.008	up
Glycolithocholic acid	2.000	0.014	up
PS(16:0/16:0)	1.059	0.016	up
Ser-Leu	1.088	0.040	up
Scytalone	−1.594	0.002	down
3-Methylglutaric acid	−1.168	0.002	down
Inosine	−1.139	0.002	down
Phenylpyruvate	−1.350	0.002	down
L-Alanine	−1.481	0.003	down
Androsterone sulfate	−3.303	0.005	down
Uracil	−1.999	0.009	down
Hydroxyisocaproic acid	−1.368	0.010	down
3,3-Dimethylglutaric acid	−1.628	0.011	down
L-Ascorbic acid	−1.754	0.012	down
N6-Methyladenine	−2.191	0.012	down
Oxyquinoline	−1.066	0.014	down
DL-lactate	−2.033	0.015	down
Thymine	−1.461	0.015	down
Actinonin	−1.112	0.015	down
Acetyl-DL-Leucine	−2.012	0.016	down
D(−)-beta-hydroxy butyric acid	−2.028	0.017	down
N-Acetyl-L-phenylalanine	−1.615	0.021	down
Acetylglycine	−1.234	0.023	down
Hydroxyphenyllactic acid	−2.060	0.024	down
Dihydroxyfumarate	−1.060	0.024	down
2-Methyl-3-hydroxybutyric acid	−1.721	0.024	down
3,3′,4,5′-Tetrahydroxy-trans-stilbene	−2.141	0.025	down
5-Hydroxyhexanoic acid	−1.017	0.029	down
4-Hydroxycinnamic acid	−1.384	0.029	down
2-Dehydro-3-deoxy-D-gluconate	−2.691	0.029	down
L-Leucine	−1.405	0.030	down
Butabarbital	−1.298	0.032	down
Cortisol 21-acetate	−4.483	0.032	down
3-Dehydroshikimic acid	−1.347	0.035	down
L-Asparagine	−1.695	0.036	down
L-Dopa	−1.049	0.038	down
Methacholine	−1.009	0.040	down
1-Methylxanthine	−1.042	0.043	down
Pseudouridine	−2.022	0.043	down
Phenol	−1.349	0.046	down
**CM vs. EM**			
Val-Gln	1.131	0.002	up
L-Alanine	1.627	0.002	up
Phenylpyruvate	1.286	0.003	up
Scytalone	1.468	0.003	up
Procaine	1.414	0.004	up
trans-3-Coumaric acid	1.018	0.006	up
Androsterone sulfate	2.677	0.008	up
2-Hydroxyadenine	1.709	0.009	up
Hydroxyisocaproic acid	1.360	0.010	up
Thymine	1.661	0.010	up
DL-lactate	2.219	0.012	up
Acetyl-DL-Leucine	2.240	0.012	up
N6-Methyladenine	2.128	0.013	up
Oxyquinoline	1.049	0.014	up
L-Ascorbic acid	1.570	0.016	up
D(−)-beta-hydroxy butyric acid	2.091	0.016	up
Acetylglycine	1.353	0.017	up
Hydroxyphenyllactic acid	2.276	0.020	up
2-Methyl-3-hydroxybutyric acid	1.866	0.020	up
Deoxygalactonojirimycin	1.028	0.021	up
L-Asparagine	2.027	0.024	up
Acetohydroxamic acid	1.187	0.025	up
Tyramine	1.023	0.026	up
4-Hydroxycinnamic acid	1.377	0.028	up
2-Dehydro-3-deoxy-D-gluconate	2.690	0.030	up
Uracil	1.398	0.030	up
Pseudouridine	2.395	0.032	up
5-Methylcytosine	1.789	0.033	up
Cortisol 21-acetate	4.326	0.033	up
L-Dopa	1.091	0.034	up
Pyridoxine	1.023	0.034	up
5-Amino-4-carbamoylimidazole (AICA)	1.201	0.039	up
N-Acetyl-L-phenylalanine	1.196	0.047	up
N2-Acetyl-L-ornithine	1.419	0.048	up
Gaboxadol	1.807	0.049	up
1-Stearoyl-sn-glycerol 3-phosphocholine	−1.187	0.007	down
p-Acetamidophenol (Acetaminophen, Tylenol)	−1.082	0.008	down
4,6-Diamino-5-formamidopyrimidine	−1.082	0.009	down
Cytidine	−1.003	0.012	down
1-Palmitoyl-sn-glycero-3-phosphocholine	−1.392	0.015	down
D-Fructose	−1.457	0.018	down
PS(16:0/16:0)	−1.175	0.019	down
7-Oxocholesterol	−1.075	0.020	down
Itaconic acid	−1.694	0.020	down
1-Stearoyl-2-hydroxy-sn-glycero-3-phosphocholine	−1.260	0.021	down
Docosatrienoic Acid	−2.023	0.025	down
Stearoylcarnitine	−2.210	0.027	down
**CM vs. FE**			
Val-Gln	1.193	0.012	up
Actinonin	−1.427	0.014	down
Itaconic acid	−1.752	0.018	down
N-Tigloylglycine	−1.774	0.018	down
6-Mercaptopurine	−1.579	0.033	down
Butabarbital	−1.725	0.041	down
sn-Glycerol 1-phosphate	−1.067	0.042	down

## Data Availability

The data are included in the article or Supplementary Material.

## Supplementary Materials

The following supporting information can be downloaded at https://www.mdpi.com/article/10.3390/ani14152164/s1. Supplementary Table S1: Composition and analysis of diet for fattening pigs; Supplementary Table S2: Metabolite annotation and functional classification; Supplementary Table S3: Metabolic pathway enrichment based on KEGG database.
