# Supplementary material for: Effect of Sex on Intestinal Microbial Metabolites of Hainan Special Wild Boars
Source: Animals (Basel). 2024 Jul 25;14(15):2164. doi: 10.3390/ani14152164 (PMC11310994; doi:10.3390/ani14152164)
Supplement: Supplementary file 1 [file animals-14-02164-s001.zip › Supplementary Table S1.pdf]

Supplementary Table S1. Composition and Analysis of Diet for fattening Pigs

Items	Diet
**Ingredients（%）**	
Corn	27.00
Soybean meal	16.00
Cassava	30.00
Wheat bran	8.00
Rice bran	15.00
Premix	4.00
Total	100.00
**Nutrient levels**	
Crude protein (%)	14.01
Digestible energy (Mcal/kg)	3.46
Crude fat (%)	1.84
Crude fiber (%)	7.45
Crude ash (%)	8.17
Calcium (%)	0.52
Available phosphorus (%)	0.43
NaCl (%)	0.51

Note: Fattening pig feed is produced by Haikou Shuangbaotai Feed Co., Ltd.
